# Implementation and performance of a fiber-coupled CMOS camera in an ultrafast reflective high-energy electron diffraction experiment

**DOI:** 10.1063/4.0000284

**Published:** 2025-03-06

**Authors:** Jonas D. Fortmann, Alexander Kaßen, Christian Brand, Thomas Duden, Michael Horn-von Hoegen

**Affiliations:** 1 Faculty of Physics, University of Duisburg-Essen, 47057 Duisburg, Germany; 2 Th. Duden Konstruktionsbüro, Mustangweg 17, 33649 Bielefeld, Germany; 3 Center for Nanointegration (CENIDE), University of Duisburg-Essen, 47057 Duisburg, Germany

## Abstract

The implementation of a monolithic fiber-optically coupled CMOS-based TemCam-XF416 camera into our ultra-high vacuum (UHV) ultrafast reflection high-energy electron diffraction setup is reported. A combination of a pumpable gate valve and a self-built cooling collar allows UHV conditions to be reached without the need to remove the heat-sensitive device. The water-cooled collar is mounted to the camera housing and prevents heating of the camera upon bakeout of the UHV chamber. The TemCam possesses an one order of magnitude higher spatial resolution, which provides 30% higher resolution in reciprocal space than the previously used microchannel plate detector. The low background intensity and the four times larger dynamic range enable analysis of the diffuse intensity of the diffraction pattern like Kikuchi lines and bands. A key advantage over the previous MCP detector is the complete absence of the blooming effect, which enables the quantitative spot profile analysis of the diffraction spots. The inherent light sensitivity in an optical pump experiment can be overcome by subtracting a pump image without a probe, using photons with hν<1.12 eV (indirect bandgap of silicon), or shielding any stray light.

## I. INTRODUCTION

With the commercial availability of modern imaging electron detectors with high pixel resolution for application in transmission electron microscopes, there is growing interest in using such cameras for studies of the ultrafast dynamics in condensed matter. They promise a much higher detective quantum efficiency (DQE) than the traditional/well-established multichannel plate detectors (MCP). Such, almost each of the scarce electrons in ultrafast diffraction or microscopy experiments contributes to the image as low repetition rates in pump–probe setups, minimization of space charge repulsion by a low number of electrons per pulse or by single electron pulses,^1^ and a small scattering cross section of samples require high detection efficiency.

In monolithic direct electron detectors, electrons directly generate electron–hole pairs in a Si-based CMOS chip. These detectors can be operated in the counting mode and are therefore capable of background-free detection of single electrons in ultrafast transmission electron diffraction (UTED) and transmission electron microscopy (UTEM) experiments.^2–5^

Indirect monolithic fiber-optically coupled CMOS-based detectors utilize a thin polycrystalline phosphorus scintillator as the electron-sensitive component. High-energy electrons can pass the thin aluminum cover layer and subsequently generate so many photons in the scintillator that, at sufficient primary electron energy events on the CMOS chip, can be assigned to single electrons.^6,7^ Such cameras are operated successfully for electron diffraction,^8,9^ microscopy,^10–21^ and spectroscopy.^22^

Here, we report on the upgrade of our ultrafast reflection high-energy electron diffraction experiment (URHEED)^23–28^ from an MCP detector (Burle Chevron 3040FM) to a fiber-optically coupled CMOS-based ultra-high vacuum (UHV)-compatible camera (TVIPS TemCam-XF416 UHV).^29^

Employing URHEED, we study the structural dynamics at the surface of crystalline samples subsequent to impulsive excitation through a fs-laser pulse. Since such experiments require clean surfaces without contamination by adsorption from residual gas, ultrahigh vacuum conditions in the range of $1\times 10^{-10}$ mbar are mandatory. Otherwise, the surface structure can be significantly affected within an hour or less. As the TemCam-XF416 UHV detector is, to our knowledge, the only commercially available imaging electron detector for 30 keV electrons that is UHV-compatible, we decided to use this product. Since the fiber decouples the scintillator from the CMOS device and its readout electronics, degassing of non-UHV-compatible components is excluded for the TemCam.

However, even this camera cannot be baked-out, as is required to obtain UHV conditions. Therefore, we developed and tested a scheme for bakeout, which guarantees UHV conditions and avoids venting of the camera in the case of venting the UHV-apparatus. In addition, we characterize its performance in comparison to the previously used MCP detector.

## II. DETECTOR INTEGRATION AND HANDLING

While the TemCam is compatible with UHV conditions, the challenge is to achieve these, as such cameras must not get hotter than 40 °C under any circumstances, otherwise serious damage can occur. Establishing UHV conditions, however, requires prolonged bakeout of the entire chamber to temperatures well above 100 °C, which is incompatible with the maximum temperature requirements of the camera. A possible solution may be to dismount the camera before bakeout but involves considerable drawbacks.^10^ The camera may be physically damaged during assembly and reassembly. It is exposed to ambient conditions and moisture that enter the UHV chamber after reconnecting the camera. Upon dismounting and re-attaching the camera, the warming-up and cooling-down cycles introduce thermal stress to the fiber-CMOS unit that should be avoided. With the help of a self-built water-cooled collar we cool the housing of the camera and thus permit the bakeout of the entire chamber without dismounting and warming-up of the camera.

**FIG. 1.**
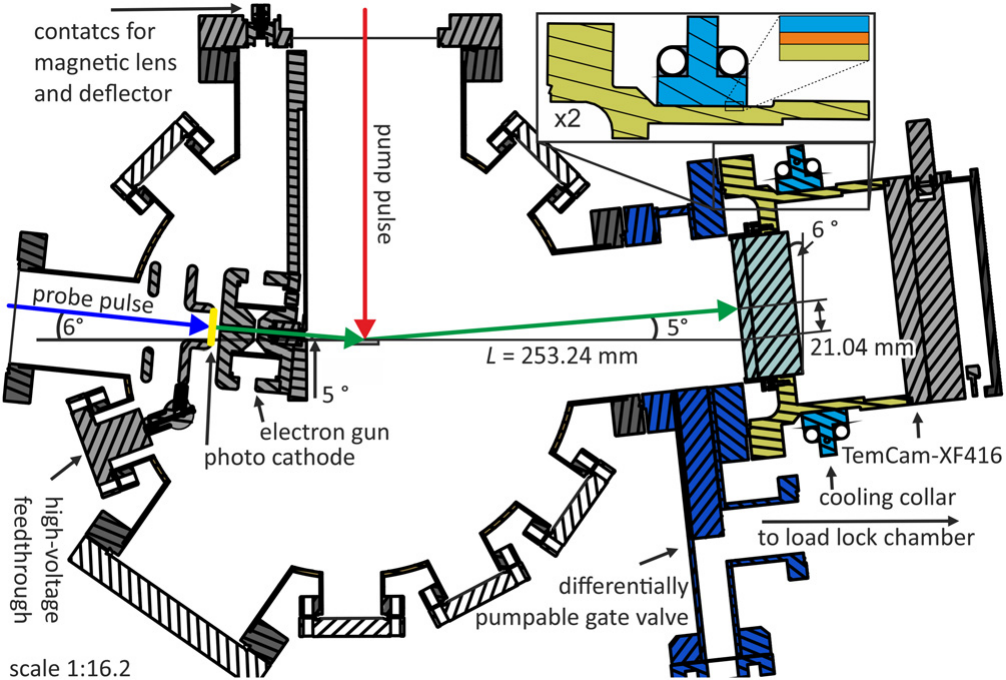
URHEED setup as cross section, with magnification of the cooling collar in the top right. Dark blue: differential pumpable gate valve, yellow: camera housing, light blue: cooling collar, gray: hard solder capillary, and orange: copper tape.

In our setup shown in Fig. 1, the camera is mounted to the main chamber (MC) via a gate valve (VAT CF-F 100 CF-F 160). The gate valve exhibits an additional CF-F 40 flange on the camera side with an angle valve (VAT 28.4 CF-F 40) attached, which is connected to a turbo molecular pump (Pfeiffer HiPace 80) at the load lock chamber (LL) with a base pressure better than $3\times 10^{-8}$ mbar. This combination allows the camera to be pumped through the MC or connected to the LL without mutual interference. During imaging operation of the camera the angle valve to the LL is closed. The MC of the URHEED experiment is pumped by a turbo molecular pump (Pfeiffer TMU 521), an ion getter pump (Varian), and two getter pumps (SAES CapaciTorr^®^-D 400-2). The pressure is measured by means of an ion gauge (MKS Granville Phillips GP307).

In the case of venting the MC, the gate valve is closed, the camera is kept cold via its internal Peltier cooler, and it is pumped by the LL. Exposure to ambient conditions is thus avoided. While the gate valve is heated during bakeout to ensure that UHV conditions are reached, the camera is located outside the bakeout oven and is not heated by radiation or convection. During bakeout, the gate valve is closed and thus protects the scintillator surface of the camera from any direct heat radiation.

To prevent a temperature increase of the camera by heat conducted trough the hot gate valve, we mounted the water-cooled collar on the camera housing acting as a heat sink. This collar is made of two parts of solid stainless steel half-rings, as shown in Figs. 1 and 6. Each half-ring is 30 mm wide and 10 mm thick. They are cooled by a capillary (stainless steel tube of 10 mm outer and 9 mm inner diameter), hard soldered for maximizing the thermal conductivity. Both capillaries are connected with PVC fiber-reinforced hose pipes in one single loop to the cooling water (15 °C, 1.5 l/min).

**FIG. 2.**
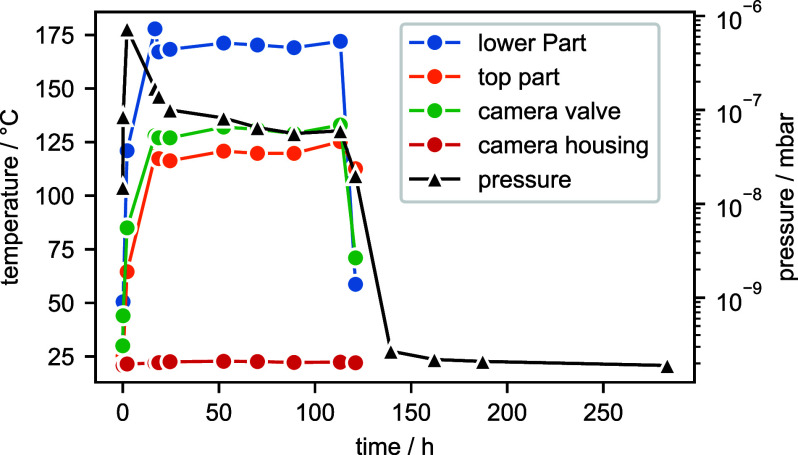
Temperature and pressure evolution during bakeout. The temperature of the gate valve separating MC and camera was measured on the flange toward the MC.

The inside of each half-ring is covered with heat conducting copper tape to maximize the thermal contact across the 139.5 ${\text{cm}}^{2}$ contact area to the camera housing (illustrated in the magnification in Fig. 1). Both half-rings are tightly connected to each other through two M4 screws (tightened at 4 Nm torque) and thus pressed onto the camera housing. K-type thermocouples, which were attached at various points on the MC, were used to monitor the temperatures during the bakeout shown in Fig. 2. The temperature of the camera housing (red data points and solid line) rises just one Kelvin from 21 to 22 °C while the temperature of the gate valve connected to the camera increases up to 130 °C. The low temperature rise by 1 K proves the efficiency of our cooling collar. The heating of the lower MC together with the ion getter pump and turbo molecular pump was turned off after 5 days, while the upper part of the MC followed 12 h later. Subsequently, the pressures dropped from $6\times 10^{-8}$ mbar to below $2\times 10^{-10}$ mbar (black triangles and line in Fig. 2. The camera was reconnected to the MC by opening the gate valve and closing the angle valve to the LL one day after bakeout.

In order to remove noise and camera artifacts—originating from the coupling of the two fiber bundle at the vacuum junction and from the CMOS sensor—from images, dark- and flatfielding is required. While dark images can be recorded for all relevant exposure times by just averaging over images taken without any electron beam, flat images require a homogeneous illumination of the whole scintillator in order to work properly. In TEM or LEEM, typically the electron source and the electron optics under strong defocusing conditions are used to homogeneously illuminate the detector.^10^ In our URHEED setup, however, the single magnetic lens was dimensioned for a small electron focal point on the detector and is unsuitable for strong de-focusing of the beam. To solve this problem, we have mounted an additional electron disk emitter (Kimball Physics ES-535), which can be brought to the sample position from the side by means of a linear translation feedthrough (VAb LDK40-150). We operate the emitter at 2.96 A biased at high negative voltage of 11 kV to maximum 20 kV in order to expose the camera with high-energy electrons. Because the emitted currents from such emitters can be quite substantial, the emission x-rays have to be monitored and controlled by, e.g., lead glass covers on the chamber windows. At emitter voltages exceeding 20 kV, x-ray fluorescence could pose an additional challenge. The emitter used in this work has an activation layer to reduce its work function. The electron beam emerging from this setup is purely divergent and thus delivers a diffuse, magnified image of the emitter surface. Because of the granular structure of the activation layer, the beam has therefore an inhomogeneous emission profile. To furthermore improve the homogeneity of the flat image, we averaged out any spatial inhomogeneity of the emitted electrons by deflection through AC magnetic fields. Two pairs of parallel coils were placed at the outside of the chamber between the disk emitter and the gate valve for deflection in horizontal and vertical direction. The coils were fed with AC generated from a function generator (Joy-IT JDS6600) and amplified with a reference audio amplifier (Behringer A800). All relevant parameters are summarized in Table I. While at constant electron emission from the disk source, we varied the exposure time in the range from 100 to 1000 ms in order to take flat images with different mean intensities. The resulting flat images only exhibit a maximum intensity inhomogeneity of ±10%, which we considered sufficient for the correction of imaging artifacts.

**TABLE I.** Parameters for the magnetic coils used for smoothing and distributing the electron pattern emitted from the disk emitter to obtain homogeneous illumination for flatfielding.

		Horizontal	Vertical
Coil	Number of windings	48	72
Signal	Waveform	CMOS	CMOS
	Amplitude (V)	2.4	2.1
	Offset (V)	0.0	0.0
	Frequency (Hz)	130	90
	Amplification (dB)	−5	−3
Measured	Coil radius (cm)	15	13.5
	Interaction length (cm)	15	11
	Maximum current (A)	2.9	3.6
Estimated	Magnetic flux (mT)	∼ 0.8	∼ 1.7
	Deflection on detector (cm)	±7	±13

## III. DETECTOR PERFORMANCE

In order to evaluate the performance of the TemCam used in our URHEED experiment, we compare it with the previously used MCP detector.^23,24,26,28^

For the TemCam a DQE(0)=0.62 (Ref. 14) with a filling factor of 0.72 (Ref. 6) is reported. For less than 1000 electrons per electron pulse, the signal-to-noise ratio is close to the shot noise limit.^7^ In contrast, the DQE of the MCP is smaller, as the channels cover only 55% of the MCP surface (filling factor of 0.55) and the probability to generate an electron cascade by multiplication in general decreases with electron energy.^30–32^

The TemCam features a square-shaped detection area with dimensions of $63.5\times 63.5\;{\text{mm}}^{2}$, comprising 4096×4096 pixels and exhibiting a 16 bit dynamic range. The MCP was imaged by a cooled CCD camera (pco.1600, PCO) with 14 bit dynamic range and 1600× 1200 pixels, with the diameter of the MCP image corresponding to 750 pixels. Especially for diffraction experiments in which the intensities of the signals in the diffraction patterns differ by several orders of magnitude, a factor four times larger dynamic range also allows weak signals to be measured without overexposing the strong spots.

A trivial but very valuable advantage of using such a fiber-coupled CMOS cameras in a diffraction experiment is its insensitivity to overexposure by electrons. In contrast, an MCP experiences severe damage upon overexposure, which easily occurs for strong diffraction spots or illumination through the direct electron beam. This robustness to damage renders it possible to characterize the direct beam without reducing its intensity, which otherwise could change properties like the beam focus. With a deflector in the electron gun the electron beam can be directed onto the TemCam and only the integration time needs to be reduced such that the image of the direct beam is not overexposed.

**FIG. 3.**
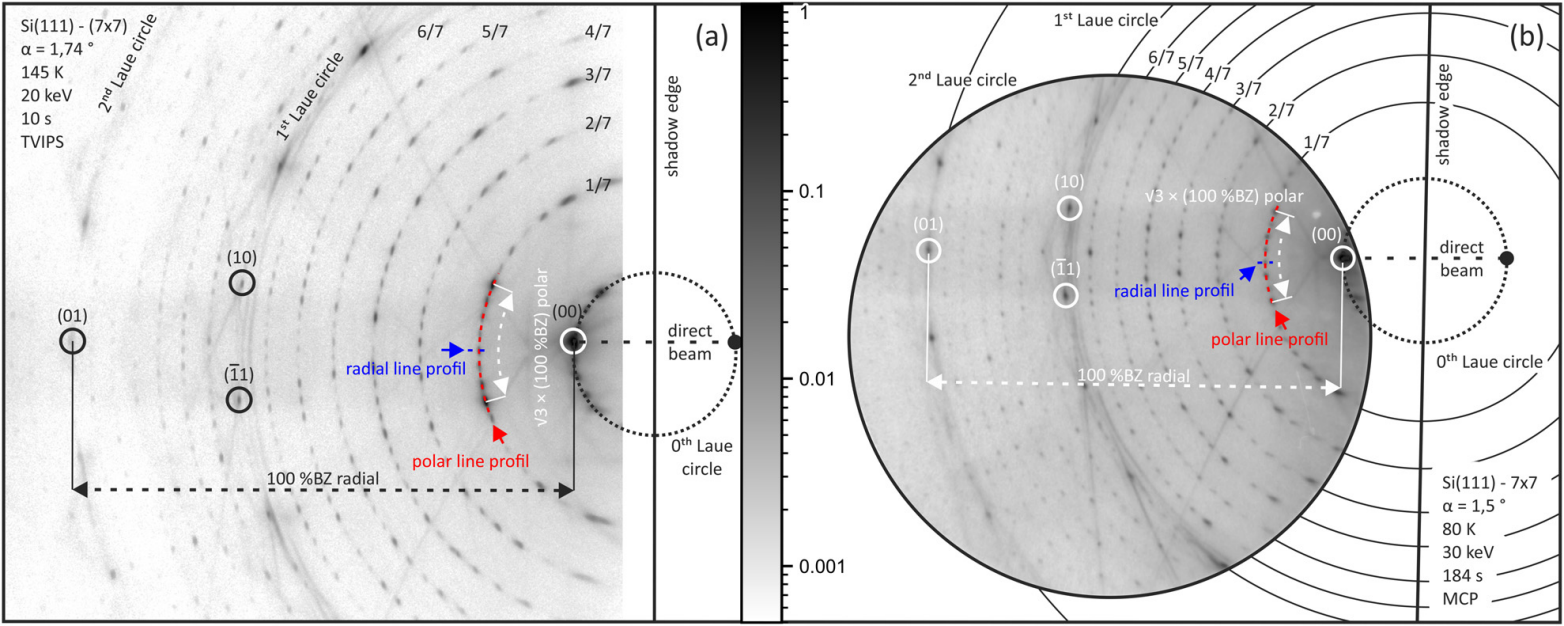
RHEED image of the Si(111)-(7 × 7) reconstruction taken with (a) the TemCam and (b) with the MCP detector. Both patterns are normalized using the same logarithmic gray scale representation. The images are scaled so that the ratio of the diameters of the zeroth Laue circles correspond to the ratio of the doubled angles of incidence.

To further evaluate the performance of the TemCam, we employed the diffraction pattern of the Si(111)–(7 × 7) reconstruction as a reference system. An image of the pattern taken with the TemCam is shown in Fig. 3(a). The exposure time was 10 s, the dark and flatcorrection was applied and it was taken with a 500 μm sized electron spot at 20 keV under an incidence angle of 1.74° and at a sample temperature of 145 K.

For comparison, Fig. 3(b) displays a diffraction pattern taken from the Si(111)–(7 × 7) reconstruction using the MCP detector.^26^ This image was acquired through summing up 92 exposures, each 2 s long, and taken with a 310 μm sized electron spot at 30 keV under an incidence angle of 1.5° at a sample temperature of 80 K. The minimum quality diameter of the circular MCP is 40 mm with a channel diameter of 10 μm and center-to-center spacing of 12 μm.

Both diffraction patterns can be compared directly as they are normalized to their maximum intensity, so they share the same logarithmic gray scale representation and display the same scale in (reciprocal) angle space. Due to the 1.79 times larger detection area, the TemCam covers a much larger fraction of the diffraction pattern and more spots become visible.

**FIG. 4.**
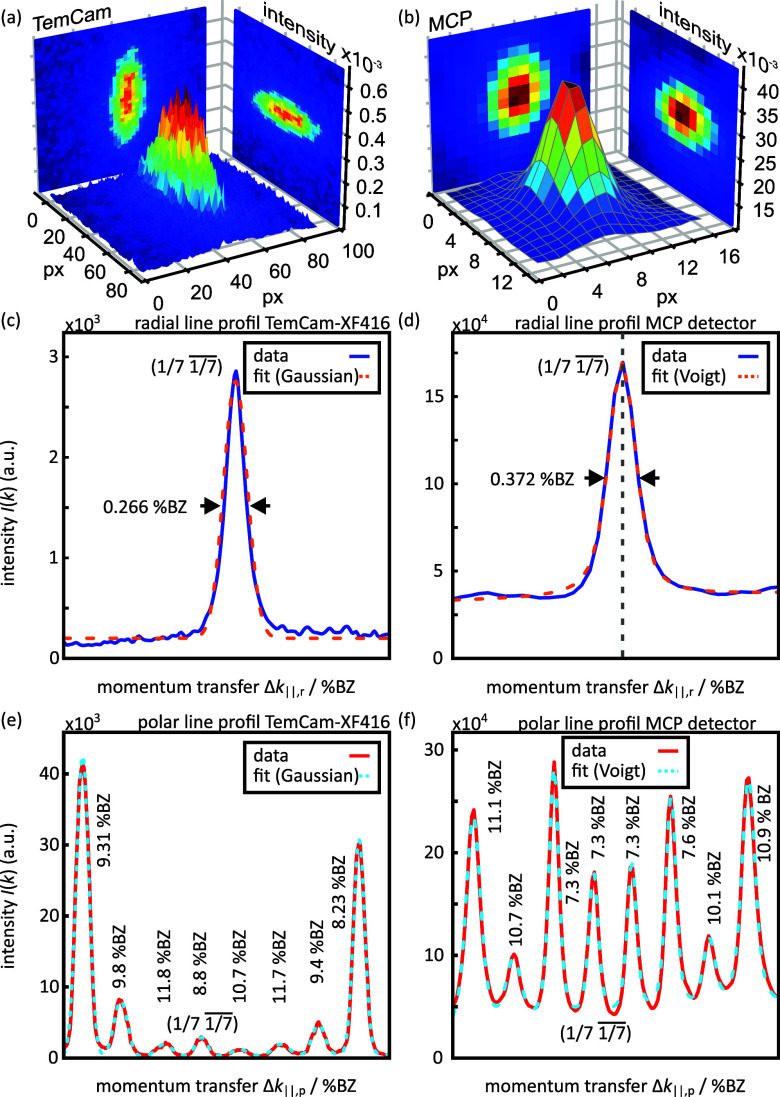
Resolution of the TemCam compared to the MCP detector. The (1/7 $\bar{1/7}$) spots of the RHEED images shown in Fig. 3. are plotted in a 3D representation in (a) for the TemCam and (b) for the MCP detector. The blue line profiles (c) and (d) are taken in the radial direction from this (1/7 $\bar{1/7}$) spot, and the red line profiles (e) and (f) in the polar direction along the 1/7th Laue circle as marked in blue and red in both RHEED images in Fig. 3.

Due to the higher pixel density, each diffraction spot was imaged with a larger number of pixels. For the MCP and for the TemCam each pixel of the image corresponds to 2844 and 240 $\mu m^{2}$, respectively. This difference of a factor of twelve in the spatial resolution is clearly visible in Fig. 4. The (1/7 $\bar{1/7}$) spot of each of the two patterns is shown in (a) and (b): the difference is remarkable. The elongated elliptical shape of the spot, as measured with the TemCam, was not resolvable with the MCP due to the limited number of pixels and the inherent blooming effect.^33^ Intensity line profiles in radial Figs. 4(c) and 4(d) and polar (e) and (f) direction enable a comparison of the resolution in reciprocal space in both directions. The scales of the axes were calibrated using the distance of first-order spots in each direction and corrected for the nonlinearity of the RHEED geometry.

For the TemCam, a Gaussian profile with a FWHM of 0.266%BZ (100%BZ = 1.89 ${Å}^{-1}$) corresponding to 0.0043 ${Å}^{-1}$ describes the profile in a radial direction. For the MCP detector, a Voigt profile with a FWHM of 0.372%BZ (0.0070 ${Å}^{-1}$) is more suitable and is explained by the lower number of pixels and the blooming effect. The FWHM corresponds to transverse coherence lengths in a radial direction of $\xi_{r,\text{TemCam}}=125$ nm for the TemCam and $\xi_{r,\text{MCP}}=89$ nm for the MCP detector.

The intensity profiles through a series of (7 × 7) spots along the polar direction are shown in Figs. 4(e) and 4(f). The difference in relative intensities is explained by the different scattering conditions, i.e., different angles of incidence and energy of the electrons ( Δα=0.27°). Nevertheless, the FWHM of all spots are in a comparable regime. For the (1/7 $\bar{1/7}$) spot, the resulting transverse coherence lengths in polar direction are $\xi_{p,\text{TemCam}}=3.8$ nm for the TemCam and $\xi_{p,\text{MCP}}=4.5$ nm for the MCP detector. The detrimental influence of the low number of pixels and the blooming effect of the MCP detector becomes obvious along the polar direction: the wings of all spots merge into one another.

Even after fitting Voigt profiles to the spots for the MCP detector, a residual background of 0.4% of the maximum spot intensity remains. This high background level originates from CCD camera-related thermally induced background, which has been subtracted—the level of shot noise, however, still contributes to the diffraction image. As a second contribution, the inherent high noise level of the MCP-unit is not subtracted, because no dark images are available. For the TemCam, the residual background is only 0.1% of the maximum spot intensity.

With the TemCam, all (7 × 7) spots can be described well with Gaussian profiles. As no blooming effect is present, the wings of the spots decay without merging into one another. While a quantitative comparison of the relative intensities is difficult due to the different scattering conditions, we observe a qualitative difference in the signal-to-background ratio of 530:1 and 12:1 for the TemCam and MCP detector, respectively.

**FIG. 5.**
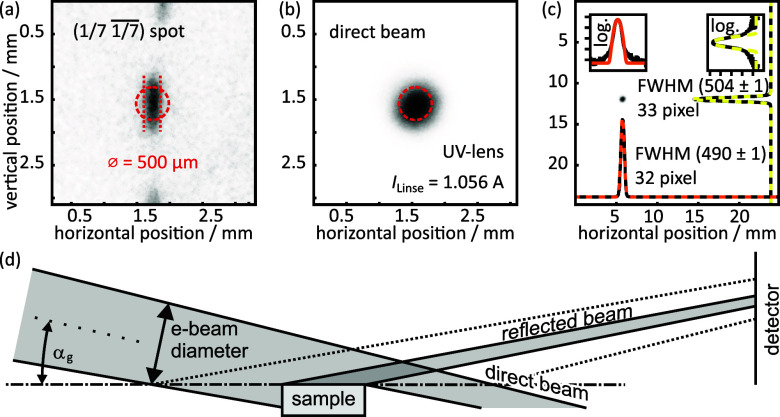
Influence of the sample width on the spot shape. (a) The (1/7 $\bar{1/7}$) spot from the image taken with the TemCam displayed in units of millimeter as imaged on the camera. (b) Spot of the focused direct beam, imaged with 100 ms exposure time. Same scale as in (a). (c) Direct beam, zoomed out by a factor of eight. Orange and yellow dotted lines are Gaussians fitted to the horizontal and vertical line profiles, respectively. Small insets display them in logarithmic scale to show the good agreement. (d) Scheme of the beam path illustrating that the narrow sample limits the angular distribution of the electron beam in one dimension resulting in a higher radial resolution.

**FIG. 6.**
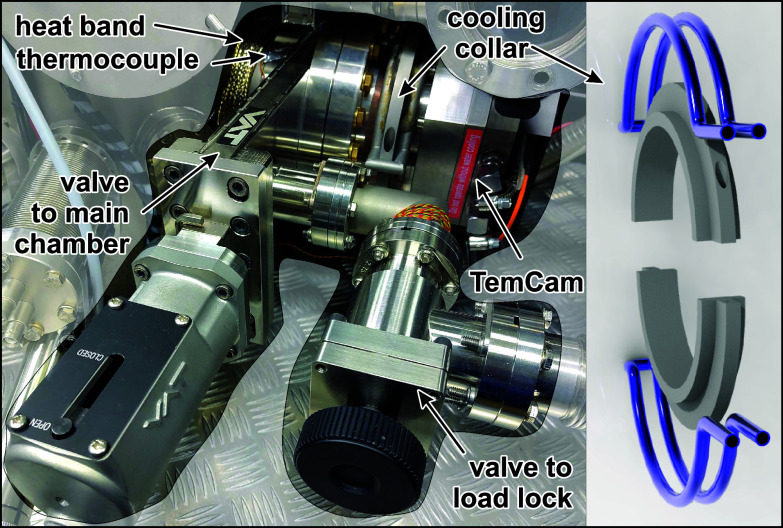
Our setup depicting the cooling collar (exploded view in the right panel), attached directly on the TVIPS TemCam-XF416, which is mounted via a VAT gate valve CF-F 100 CF-F 160 at the main chamber. On the camera side an additional CF-F 40 pump flange is connected to our load lock system through an angle valve. On the side of the main chamber a heating tape is located and a type K thermocouple is installed in order to monitor the temperature.

Inspection of the spots on the 1/7 Laue ring at higher magnification [see Fig. 5(a)] reveals a surprising finding. The (7 × 7) spots exhibit a larger width along the polar direction as compared to the width along the radial direction. For diffraction from a perfect surface, one would expect a circular spot^34^ at a width given by the focus properties of the electron gun—for the (00)-spot in RHEED, the sample principally acts as a mirror. However, due to defects at the surface, like steps, domain boundaries, etc., all spots experience additional broadening.^35,36^ Given the higher transverse coherence length along the radial direction, the broadening should be more pronounced in this direction. Here, however, we observe the opposite!

The direct electron beam is shown in Figs. 5(b) and 5(c) and exhibits the expected Gaussian-shaped circular spot profile with a width, which agrees with the spot width in polar direction. In general, the width of the direct beam is determined by the electron source size at the photocathode, the magnification during imaging onto the detector, and spherical aberrations of the single magnetic lens.^26^

We attribute the smaller spot width in the radial direction to the exclusion of off-axis beams that experience large spherical aberrations from the image construction. In RHEED, the grazing incidence of 1°–6° ensures surface sensitivity.^37^ Here, the electron beam is incident at a grazing angle of $\alpha_{g}=1.74$° with respect to the sample surface. This results in a so-called foreshortening factor of $1/\;\text{sin}(\alpha_{g})≃33$, i.e., the beam size on the sample position is elongated by a factor of 33.

Thus, for a finite beam diameter of ≃300 μm the sample acts as a narrow slit of 1.7 mm/33 = 0.05 mm width for the image construction of the diffraction pattern as sketched in Fig. 5(d). In the radial direction, only the central on-axis beams contribute to image formation. The off-axis beams that experience large spherical aberrations are suppressed from image formation, because they are either blocked or do not hit the sample. Consequently, the spots become sharper in this direction with a superior transverse coherence length of $\xi_{r,\text{TemCam}}=125$ nm.

## IV. CONCLUSIONS

We have demonstrated that with the help of a water-cooled cooling collar and a differential pumpable gate valve, the bakeout of temperature-sensitive equipment like the TemCam is possible without dismounting the camera.

By this upgrade from an MCP detector to the TemCam we improved the resolution in reciprocal space by 30%. Thus, we are able to perform spot profile analysis without MCP-related blooming effects and at nine times higher pixel density. The low background intensity of the TemCam enables to study weak features in the diffraction pattern such as Kikuchi lines and bands that were previously inaccessible in our URHEED experiment.

One drawback we have to accept for measurements with the TemCam at full resolution is the inherent dead time of 1.2 s due to the readout of the 4k CMOS chip. For adjustment and optimization, however, the TemCam is fast enough at 10 Hz in continuous rolling shutter mode at full resolution. Also, the four times higher dynamic range allows for measuring analyzable data of weak and strong diffraction signals simultaneously, which reduces the overall measuring time.

Due to its scintillator/CMOS chip-based design, the TemCam is inherently sensitive to photons with an energy above the indirect bandgap of Si. This must be taken into account when the TemCam is employed in experiments with optical excitation. When pumping with wavelength of 800 nm photons from a Ti:sapphire laser, effective shielding of the exciting pump laser pulse must be considered. Alternatively, photons with an energy $h\nu <E_{\text{gap}}=1.12$ eV can be used as pump pulse. As a first step for correction, an image of the stray light of the pump without probing electrons can be used for subtraction from the diffraction pattern. The light sensitivity can be lower for setups with higher electron energies. Then the aluminum layer covering the scintillator can be thicker than the 30 nm, which thickness in our case is optimized for the detection of 30 keV electrons.

Further improvements of the signal-to-noise ratio in an electron counting mode may be achieved by direct electron detectors, as their DQE is reported to be even higher than for the TemCam.^12,14^ However, direct electron detectors, which are designed for electron energies of 30 keV or less, are not yet available in a dedicated UHV-compatible version and also suffer from unintended light sensitivity at 800 nm.

In contrast, the design of the TemCam may allow placing an interferometric filter between the scintillator and the CMOS chip, thus blocking the 800 nm pump light from being detected. A major advantage in the daily operation of the TemCam, however, is its insensitivity and robustness to destruction caused by overexposure in the case of intense diffraction spots or by the direct electron beam.

## Data Availability

The data that support the findings of this study are available from the corresponding author upon reasonable request.

## APPENDIX: IMPLEMENTATION IMAGE

A labeled photograph of our UHREED setup as well as a rendering of the cooling collar is shown in Fig. 6.
